# Peripheral artery disease: an underdiagnosed condition in familial hypercholesterolemia? A systematic review

**DOI:** 10.1007/s12020-024-03763-x

**Published:** 2024-03-08

**Authors:** Elisa Acitelli, Alexis F. Guedon, Sara De Liguori, Antonio Gallo, Marianna Maranghi

**Affiliations:** 1 Department of Translational and Precision Medicine, Sapienza University of Rome, Rome, Italy; 2 Département Hospitalo-Universitaire Inflammation Immunopathologie Biothérapie (DMUi3), Sorbonne Université, APHP, Service de Médecine Interne, Paris, France; 3 Department of Nutrition, APHP, Hôpital Pitié-Salpètriêre, Sorbonne Université, INSERM UMR1166, Lipidology and cardiovascular prevention Unit, 47/83 boulevard de l’Hôpital, F-75013 Paris, France

**Keywords:** LDL-cholesterol, Atherosclerosis, Dyslipidemia, Peripheral vascular disease, Hypercholesterolemia

## Abstract

**Purpose:**

Familial hypercholesterolemia (FH) is one of the most common inherited diseases characterized by elevated LDL-cholesterol levels, leading to early-onset atherosclerosis. While the association between FH and coronary and carotid artery disease is well-established, its association with peripheral artery disease (PAD) is less robust. This systematic review aims at exploring existing evidence on PAD prevalence and incidence in FH individuals.

**Methods:**

A comprehensive search was conducted on MEDLINE and Embase databases, for studies published between January 2013 and December 2023, evaluating prevalence and incidence of PAD in FH patients. Literature reviews, case reports, responses to editors and non-English language articles were excluded.

**Results:**

The initial research provided 53 results. After article screening, 28 articles were fully reviewed and 24 were finally included in the analysis. Among these, 19 reported PAD prevalence, while 5 PAD incidence over a mean follow-up time of 8.7 years. PAD prevalence and incidence ranged from 0.3 to 60% and from 0.5 to 4.2% respectively, probably reflecting the heterogeneity in PAD definition criteria.

**Conclusion:**

This systematic review sheds light on the limited number of studies on PAD in FH patients. Particularly, considering the potential positive effects of newly available lipid-lowering strategies on PAD outcomes, addressing this research gap is pivotal for a more comprehensive understanding of peripheral vascular manifestations in FH patients and for optimal management of this population.

## Introduction

Familial hypercholesterolemia (FH) is one of the most common autosomal dominant genetic diseases, characterized by an increased exposure to elevated low-density lipoprotein cholesterol (LDL-C) levels that result in a propensity for early atherosclerotic cardiovascular disease (ACVD) [1].

It is caused by mutations in genes involved in LDL-C catabolism, such as functional mutation in the LDL receptor (LDLR), apolipoprotein B (APOB) or proprotein convertase subtilisin kexin-9 (PCSK9) [2, 3]. The prevalence of FH in the general population is about 1 in 300, affecting about 14 million people worldwide [4, 5].

The association between FH and coronary artery disease is widely described; the prevalence of clinical FH among patients with premature acute myocardial infarction (MI) (age ≤50 years) is approximately 30 times higher than in the general population and cardiovascular risk factors prevalence increases with age of diagnosis [1, 6].

Peripheral artery disease (PAD) is a prevalent yet underdiagnosed condition characterized by a wide spectrum of clinical manifestations [7]. Only 10 to 30% of patients with PAD present with classical symptoms of intermittent claudication, while the remaining present with non-specific symptoms or are asymptomatic [7]. It is estimated to affect over 200 million adults in the world and its burden has continuously increased since 1990 [8]. Diabetes, cigarette smoking and hypertension have been associated with an increased risk of PAD, whereas the relationship between dyslipidemia and PAD is rather multifaceted, with features of atherogenic dyslipidemia being more related to PAD than simple LDL-C levels [9, 10].

Methodologies for PAD diagnosis include ankle-brachial index (ABI), segmental limb pressure evaluation, treadmill testing, pulse-volume waveform analysis and imaging techniques (such as duplex ultrasonography, CT angiography and MR angiography) [7].

The first-line noninvasive diagnostic method for PAD is the ABI, with sensitivity and specificity ranging from 61 to 73% and from 83 to 96% respectively [7]. However, sensitivity might decrease and ABI might be falsely high in patients with advanced age, diabetes or chronic kidney disease, because of the higher probability of medial artery calcification [7]. In these particular cases, other tests such as the toe-brachial index (TBI) may be considered [7].

Data from the largest international registry of FH suggest that PAD is found in about 5% of FH population [1], however it is unclear whether diagnosis is based on symptoms, imaging, or other methods like ankle brachial index (ABI). In addition to the healthcare expenses linked to addressing complications related to PAD, a primary concern is the insufficient awareness of the condition both among patients and practitioners [1].

This systematic review aims to provide a comprehensive assessment and investigate the prevalence and incidence of PAD in individuals living with FH.

## Methods

### Search strategy

The study protocol was registered on PROSPERO (registration number CRD42023476568) in accordance with PRISMA guidelines [11].

The online MEDLINE and Embase databases were searched for medical subject heading (MeSH) and keywords related to PAD and FH (see Supplementary Methods 1). These searches were supplemented by examining reference lists of included studies, reviews, and meta-analyses.

### Studies selection

Inclusion criteria were as follows: (1) randomized controlled trial (RCTs) and/or observational studies; (2) evaluating the prevalence of peripheral artery disease (PAD) in patients with familial hypercholesterolemia (FH) published between 1 January 2013 and 14 November 2023.

Literature reviews, case reports, responses to editors and non-English language articles were excluded. The initial research provided 53 results. After article screening, 28 articles were fully reviewed and 24 articles referring to heterozygous familial hypercholesterolemia (HeFH) were included in the final analysis (Supplementary Fig. 1). Among these articles, there were 9 cross-sectional studies, 7 retrospective studies, 6 prospective studies and 2 pooled randomized controlled trials (RCTs).

### Data extraction

Data extracted from selected articles included: year of publication, type of study, journal, first author, recruitment time or follow-up, population (number of patients), gender, age, FH diagnosis (molecular or clinical), ethnicity, country, baseline lipid profile, comorbidities (hypertension, diabetes, smoking), family history of premature cardiovascular disease, ACVD status (primary or secondary prevention), lipid lowering therapy, criteria for PAD definition, study outcome and results, reported prevalence or incidence of PAD, coronary artery disease (acute coronary syndrome or stable angina) and carotid artery disease (stenosis or stroke).

## Results

The main information extracted from the reviewed articles is summarized in Table 1 and Table 2. Nineteen studies reported PAD prevalence (Table 1) while 5 studies reported PAD incidence during the follow-up time (Table 2). The age distribution of study populations ranged from 40.3 to 62.7 years. Reported PAD prevalence ranged from 0.7 and 60% in cross sectional studies, from 0.3 to 15.6% in retrospective studies and from 1.4 and 12.4% in prospective studies. The 2 RCTs reported a PAD prevalence of 5.7 and 6%. Reported PAD incidence varied from 0.5 to 4.2% during a mean observation time of 8.7 years.

**Table 1:** Selected information of studies reporting PAD prevalence

Year, Journal, First Author Country	Study design	Population (n)	Age (y)	Gender (%M)	Cardiovascular risk factors (%)	Lipid-lowering therapy (%)	ASCVD prevalence (%)	PAD definition	FH definition	PAD prevalence (%)	PAD prevalence as primary outcome
2016, Arterioscler Thromb Vasc Biol, L Pérez de Isla, Spain [37]	Prospective	2 752	NA	45.9	Diabetes 3.2 Hypertension 14.4 Smoking 26.4	Statins 38 Ezetimibe 36 Max combined therapy 21.5 Max LLT 48.2	CHD 11.8 CBVD 1.8	IC and ABI < 0.9 or stenosis >50% at imaging or AAA	Genetic	1.4	Yes
2015, Atherosclerosis, C Pereira, Brazil [18]	Cross sectional	202	50.8	35	Diabetes 17.3 Hypertension 49.5 Smoking 35.2	Statins 89	NA	ABI ≤ 0.9	DLCN criteria or genetic	1.3	Yes
2022, J Am Heart Assoc, S Funabashi, Japan [38]	Cross sectional	370	56.4	43	Diabetes 2.4 Hypertension 25.4 Smoking 31	Statins 87 Ezetimibe 58 PCSK9i 16	CHD 26.8 CBVD 4.6	Self-reported IC or ABI < 0.9 or >50% stenosis at imaging	Japan Atherosclerosis Society guidelines	3	No
2021, Atherosclerosis, AJ Vallejo-Vaz, Multicenter Scandinavian countries [39]	RCT	152	51.6	62	Diabetes 3.9 Hypertension 26.3 Smoking 36.2	NA	CHD 76 CBVD 0	Claudication	DLCN criteria	6	No
2018, J Clin Endocrinol Metab, F Emanuelsson, Denmark [40]	Prospective	7 109	59.9	45	Diabetes 6.5 Hypertension 66.4 Smoking 22.5	LLT 27.7	CHD 11	ICD-8 440-441, 443.99, 445 and ICD-10 I70-I72 and I73.9	DLCN criteria	12.4	Yes
2019, J Clin Med, L Masana, Spain [41]	Retrospective	12 823	61.7	44.8	Diabetes 24.3 Hypertension 66.3 Smoking 35.2	LLT 89	CHD 46.9 CBVD 22.8	ICD-9 4439 44020-4402444029-4403244422, 44489 and ICD-10 I70, I70.2, I70.8, I70.9 I73, I73.8, I73.9 I74.3	Validated age-adjusted LDL-C thresholds	15.6	Yes
2018, Atherosclerosis, YX Cao, China [14]	Cross sectional	151	47.2	53.8	Diabetes 14.6 Hypertension 36.4 Smoking 32.5	Statins 73.5	CHD 66.9 CBVD 70.2	Femoral plaque (local enlargement ≥0.5 mm or 50% of the surrounding IMT or IMT > 1.5 mm.)	DLCN criteria or genetic	54.5	No
2019, NMCD, A Mattina, France [15]	Cross sectional	154	48.3	45	Diabetes 0 Hypertension 12 Smoking 27	Statins 78 Ezetimibe 34 PCSK9i 0 Statin+Ezetimibe 80	CHD 62 CBVD 55	Femoral plaques (c-IMT > 1.5 mm, irregular shape, wall structure alteration)/stenosis	Genetic	56	No
2017, Arterioscler Thromb Vasc Biol, AJ Amor, Spain [16]	Cross sectional	240	46	51	Diabetes 4 Hypertension 13 Smoking 22	NA	CBVD 56	Femoral plaque	Genetic	60	No
2018, Am J Cardiol, PA McCullough, Multicountry [42]	Pooled RCT	1 257	52.7	54	Diabetes 11.2 Hypertension 43 Smoking 17.7	Statins 92 Ezetimibe/other LLT 61	CHD 95 CBVD 7.6	NA	DLCN criteria or genetic	5.7	No
2022, Atheroscler Plus, WT Fonzar, Brazil [43]	Cross sectional	92	59	30	Diabetes 14 50 Smoking 14	LLT 83	CHD 17 CBVD 6	NA	DLCN criteria or genetic	3	No
2020, Oman Med J, K Al-Waili, Oman [44]	Cross sectional	439	47.6	55.8	Diabetes 17.7 Hypertension 26.4 Smoking 11.6	Low-intensity statin 12 Medium-intensity statin 41.5 High-intensity statin 46.5 Statin+Ezetimibe 20	CHD 22.3 CBVD 2.9	NA	DLCN criteria	0.7	No
2022, Endocrine, P Anagnostis, Greece [45]	Cross sectional	541	48.5	46	Diabetes 6.8 Hypertension 22 Smoking 22.4	NA	CHD 37 CBVD 5	NA	DLCN criteria	8.3	No
2017, Circ J, B Zafrir, Israel [46]	Cross sectional	1 690	49	42	Diabetes 14 Hypertension 25 Smoking 23	Statins 57	CHD 83 CBVD 19	NA	Modified MEDPED criteria	13	No
2019, Atherosclerosis, PB Duell, US [47]	Prospective	1 900	55.8	39.2	Diabetes 14.2 Hypertension 46.8 Smoking 36	Statins 73.8 PCSK9i 18.9 LDL apheresis 3.2	CHD 14	NA	Clinical (including but not limited to Simon Broome, Dutch Lipid Clinic Network, MEDPED) or genetic	5.9	No
2021, Med Sci Monit, Matta A, France [48]	Retrospective	123	59	53.7	Diabetes 7.3 Hypertension 24.4 Smoking 3.3	Statin+Ezetimibe 22.8 Ezetimibe 9.8 LLT 67.5	CHD 66.7 CBVD 2.4	NA	DLCN criteria	9.8	No
2022, Front Genet, V Todorovova, Chzechia [49]	Retrospective	1 236	44.8	31	Diabetes 6.5 Hypertension 26.7 Smoking 31.4	NA	CHD 9.6 CBVD 2.5	NA	DLCN criteria or genetic	2.6	No
2019, Turk J Endocrinol Metab, MA Eren, Turkey [50]	Retrospective	267	47.1	41.5	Hypertension 6.3	NA	CHD 12 CBVD 4.1	NA	DLCN criteria	0.3	No
2018, J Atheroscler Thromb, T Teramoto, Japan [51]	Retrospective	3 495	62.7	40.3	Diabetes 28.6 Hypertension 55.3	Low/moderate-intensity statins 45.9 High-intensity statins 2.9 Non-statin LLT 6.6	CHD 16.7 CBVD 1.2	NA	NA	6.8	No

Some data were extracted from those reported in the original articles

*NA* not available, *LLT* lipid-lowering therapy, *CHD* coronary heart disease (acute coronary syndromes, stable angina), *CBVD* cerebrovascular disease (stenosis, stroke), *IC* intermittent claudication, *ABI* ankle brachial index, *AAA* abdominal aortic aneurysm, *DLCN* Dutch Lipid Clinic Network, *IMT* intima-media thickness

**Table 2:** Selected information of studies reporting PAD incidence

Year, Journal, First Author Country	Study design	Population (n)	Follow-up (y)	Age (y)	Gender (%M)	Cardiovascular risk factors (%)	Lipid-lowering therapy (%)	ASCVD incidence during observation time (%)	PAD definition	FH definition	PAD incidence during observation time (%)	PAD incidence as primary outcome
2021, JACC Asia, S Funabashi, Japan [52]	Retrospective	380	7.4	40.3	39.5	Diabetes 1.8 Hypertension 18 Smoking 25	Statins 81 Ezetimibe 46 PCSK9i 11 LDL apheresis 1.3	CHD 31 (8.2) CBVD 11 (2.9)	Self-reported IC or ABI < 0.9 or >50% stenosis at imaging	Genetic	3 (0.8)	No
2022, Eur J Prev Cardiol, LJ Mundal, Norway [19]	Prospective	3 162	8	NA	NA	NA	NA	NA	ICD-9 440 and 443 and ICD-10 170 and 173	Genetic	40 (1.2) hospitalizations [2.2 per 1000 person-years (1.6–3.0)]	Yes
2022, Atherosclerosis, J Ferrieres, France [53]	Prospective	3 202	5	48.9	51.6	Diabetes 6.2 Hypertension 19 Smoking 26	Statins 24 Ezetimibe 3 Statin+Ezetimibe 29.2 Other LLT 2.6	CHD 296 (9.24) [18.8 per 1000 person-years] CBVD 44 (1.4) during observation time [2.79 per 1000 person-years]	Arterial embolism/thrombosis/occlusion/stenosis	DLCN criteria or genetic	47 (1.5) [2.99 per 1000 person-years]	Yes
2019, Atherosclerosis, B Iyen, UK [54]	Retrospective	14 097	13.8	42.5	46.7	Diabetes 2.2 Hypertension 5.6 Smoking 81.7^a^	Statins 7	CHD 3545 (25) [20.3 per 1000 person-years] CBVD 746 (5.4) [4.3 per 1000 person-years]	NA	DLCN or Simon Broome criteria	592 (4.2) during [3.4 per 1000 person-years (3.1–3.7)]	Yes
2018, AM Galema-Boers, J Clin Lipidol, Netherlands [55]	Prospective	821	9.5	47.4	47	Diabetes 4 Hypertension 28 Smoking 16	LLT 100	CHD 75 (9) CBVD 23 (2.8)	NA	DLCN criteria or genetic	4 (0.5)	No

Some data were extracted from those reported in the original articles

*NA* not available, *LLT* lipid-lowering therapy, *CHD* coronary heart disease (acute coronary syndromes, stable angina), *CBVD* cerebrovascular disease (stenosis, stroke), *IC* intermittent claudication, *ABI* ankle brachial index, *DLCN* Dutch Lipid Clinic Network

^a^Past + current smokers (“ever smoked”)

### PAD prevalence and incidence (according to definition criteria)

In the reviewed literature, PAD definition criteria were heterogeneous.

#### Prevalence

A subset of studies (*n* = 4) defined PAD with more stringent criteria (i.e. as intermittent claudication with either ABI < 0.9 or evidence of stenosis >50% at imaging; ABI ≤ 0.9 alone; intermittent claudication or ABI < 0.9 or stenosis >50% at imaging; claudication alone) and reported the lowest prevalence (1.3–6%).

Two studies defined PAD according to the International Classification of Diseases (ICD) system (Table 1 and Supplementary Table 3), reporting a PAD prevalence of 12.4 and 15.6%.

Three studies defined PAD according to wider criteria (evidence of femoral plaque, IMT > 1.5 mm). Among these studies, PAD prevalence was similar and ranged between 54.5 and 60%.

Finally, a substantial subset of articles (*n* = 10) did not explicitly provide a specific definition for PAD. Among these articles, PAD prevalence showed the highest variability (ranging from 0.3 and 13%).

Interestingly, the majority of studies aiming at assessing PAD prevalence as a primary outcome reported overall lower prevalence rates compared to those for which prevalence assessment was not a primary outcome.

#### Incidence

Among the 5 studies evaluating PAD incidence, one defined PAD as intermittent claudication or ABI < 0.9 or stenosis >50%: in this study the incidence of PAD was as low as 0.8% during the observation time. One study relied on the International Classification of Diseases (ICD) system (Table 2 and Supplementary Table 3) for PAD definition and reported an incidence of hospitalizations due to PAD of 1.2%.

One study, defining PAD as arterial embolism, thrombosis, occlusion or stenosis, reported an incidence rate of 1.5%.

Two studies did not specify PAD definition criteria, and reported an incidence of 0.5 and 4.2%.

In this case, a lower incidence was reported in studies for which incidence assessment was not the primary outcome.

### FH definition

The majority of studies relied on the Dutch Lipid Clinic Network (DLCN) criteria and genetic analysis, either individually or in combination. A minority of studies employed alternative clinical criteria for defining FH, such as the Simon Broome criteria, MEDPED criteria, or the Japan Atherosclerosis Society guidelines.

Moreover, clinical features that accurately characterize the FH population (including detailed lipid-lowering treatment, lipid profile and category of cardiovascular prevention) were often lacking in the analyzed studies, and are shown in the Supplementary Tables 1 and 2.

### PAD and cardiovascular risk factors

All studies evaluating PAD prevalence also reported cardiovascular risk factors prevalence including diabetes, hypertension and cigarette smoking. Diabetes prevalence ranged between 2.4 and 28.6%, with the exception of one study in which patients with diabetes were not included. Prevalence of hypertension and smoking ranged between 12 and 66.4% and between 11.6 and 45.1%, respectively. Interestingly, PAD did not vary significantly according to reported prevalence of other cardiovascular risk factors.

Four studies investigating PAD incidence reported the prevalence of other cardiovascular risk factors. Prevalence of diabetes, hypertension and smoking ranged between 1.8 and 6.2%, 5.6 and 28% and 16 and 81.7%, respectively.

Prevalence of these cardiovascular risk factors did not seem to change significantly among the studies (with the exception of one study defining smokers as both current and past smokers, thus reporting a higher smoking percentage). As outlined before, prevalence of cardiovascular risk factors was not apparently associated with a higher incidence of PAD.

### Coronary and carotid involvement

Among the 19 studies evaluating PAD prevalence, 15 investigated both coronary and carotid vascular involvement, 2 evaluated coronary involvement only, and 1 evaluated carotid involvement only. Coronary and carotid disease prevalence ranged between 9.6 and 95% and between 0 and 70.2%, respectively. In the majority of studies, prevalence of coronary and carotid diseases was higher in studies reporting a higher PAD prevalence. Four of the 5 studies investigating PAD incidence assessed incidence of coronary and carotid vascular disease as well. Coronary artery disease incidence varied from 8.2 to 25%, while carotid artery disease incidence varied from 1.4 and 5.4% during the observation time. Studies reporting the highest incidence of PAD reported the highest incidence of coronary and carotid disease as well.

## Discussion

In this review, we analyzed 24 articles reporting PAD prevalence or incidence among FH patients. Based on definition, PAD prevalence ranged from 0.3 to 60%, with the lowest variability among prospective studies and those defining PAD with more stringent criteria. The incidence of PAD during follow-up varied from 0.5 to 4.2%. Furthermore, PAD definition was not specified in 12 out of 24 articles analyzed.

Coronary and carotid involvement showed high variability in prevalence (9.6–95 and 0–70.2%, respectively) and incidence (8.2–25 and 1.4–5.4%, respectively). This high variability may reflect the fact that the majority of studies were including both subjects in primary and secondary prevention.

Regarding FH diagnosis, most studies used the DLCN criteria and genetic analysis, and detailed data on lipid-lowering treatments and lipid profiles were often missing.

Data regarding PAD epidemiology among FH patients is sparse as compared to other atherosclerotic territories involved [1]. A recent meta-analysis found a PAD prevalence of 5.2% worldwide, with a prevalence of 6.8% in Europe [1]. However, our study shows extreme variability in the prevalence estimation of PAD in FH.

The observed heterogeneity might be attributable to multiple factors. Firstly, PAD encompasses a wide range of manifestations, ranging from lower limb amputation to asymptomatic subclinical lower-limb atherosclerosis, defined by an ankle-brachial index (ABI) of 0.90 or less. Though these patients are at high risk of cardiovascular events and mortality [12], PAD is often underdiagnosed even in the general population [13]. Thus, estimating its prevalence could be difficult due to differences in the investigated populations and to the lack of a uniform PAD definition. This is clearly reflected by this systematic review. As a matter of fact, the three studies with the highest PAD prevalence (54.5, 56 and 60%) included femoral plaques in their PAD definition [14–16]; by excluding these three studies, the prevalence would range between 0.3 and 15.6% with the lowest rates reported mainly in the studies defining PAD as ABI < 0.9. Secondly, apart from PAD definition, for studies evaluating the prevalence of PAD the lower was the estimated prevalence the higher was the quality of the study design, and vice versa. Interestingly, most of the analyzed studies were conducted by clinicians alone, and only two studies were conducted by clinicians together with vascular surgeons. This may contribute to the variability of the outcome definition and account for the selection bias of the studied population. Familial hypercholesterolemia unawareness among health professionals not dedicated to lipid disorders is still quite frequent and may account for the underdiagnosis of this condition in subjects undergoing vascular procedures, highlighting the need for a multidisciplinary counseling. Thirdly, it must be remembered that about 50% of subjects with PAD are asymptomatic. Moreover, when studies evaluate PAD according to ICD definition, it must be acknowledged that the diagnostic power is reduced, even more in asymptomatic subjects.

Finally, age is one of the major PAD risk factors [9]. One systematic review estimated a global prevalence of PAD defined by an ABI of 0.90 or less in people aged 25 years and older of 5.56% (95% CI: 3.7–8.55) [17]. Another recent published metanalysis found a global prevalence of 9.7% (95% CI: 7.1–12.4) in adults mostly >40 years old, with age, smoking status, hypertension, diabetes and hypercholesterolemia ≥200 mg/dL being significantly associated risk factors [9]. In the light of these considerations, PAD prevalence among FH patients seems to be similar to the one reported in the general population. This is significant, considering that the FH population is typically younger and less exposed to cardiovascular risk factors than the general population. Lower extremity PAD is prevalent among people aged 50 years and older in the general population [9]. Though exposed to high LDL-C levels since birth, FH subjects are younger than those usually assessed in the general population. Still, age-adjusted comparisons seem to find higher PAD prevalence among FH patients than non FH patients [18, 19], suggesting a premature PAD incidence among FH patients despite the variability of PAD prevalence/incidence we analyzed. In our analysis, we could not investigate the weight of well-recognized risk factors for PAD (like smoking and diabetes), since these risk factors were not uniformly and systematically reported [20–22]. However, PAD did not seem to vary significantly according to reported prevalence of other cardiovascular risk factors when available. Familial hypercholesterolemia is clearly associated with an increased risk of coronary heart disease, as largely reported in the literature and as shown by most extreme clinical presentations, as in homozygous FH [23, 24]. However, the prevalence of common cardiovascular risk factors in this population is associated with a higher cardiovascular risk. Equations aimed at stratifying cardiovascular risk in FH clearly show an improved CV risk prediction when classical cardiovascular risk factors are taken into account [25–28]. Regarding ischemic stroke, a recent study based on the Copenhagen general population failed to show an association between FH and ischemic stroke [29], though evidence from RCTs showed a benefit on stroke from LDL-C reduction with statins [30, 31]. This illustrates the complexity of the link between LDL-C and its consequences depending on the anatomical territories assessed. Regarding PAD, increasing evidence based on Mendelian randomization studies [32] and effects from both statins and PCSK9 inhibitors on vascular outcomes tends to demonstrate a causative role of LDL-C [33–35]. Furthermore, the Progression of Early Subclinical Atherosclerosis (PESA) study has shown that lower limbs atherosclerosis in asymptomatic middle aged individuals highlight subclinical atherosclerotic burden in subjects otherwise considered at low risk [36], and femoral atherosclerosis was the best predictor of atherosclerosis in other vascular districts.

## Conclusions

The scarcity and sparsity of data available in the literature underlines the critical need for improved, more standardized and better-defined screening of PAD in patients with FH. A systematic approach in identifying PAD, either symptomatic or asymptomatic, with more uniform criteria could be useful since patients with polyvascular disease are at particularly high risk for future cardiovascular events and warrant aggressive management of standard atherosclerotic risk factors. At present, current guidelines do not recommend screening strategies for PAD, in patients without signs or symptoms of claudication. Future prospective studies evaluating PAD in subjects with FH could provide valuable insights into their long-term cardiovascular outcomes and tailor strategies of novel lipid-lowering agents such as PCSK-9 inhibitors for treatment of these high-risk individuals, in particular in the context of improved life- expectancy.

### Supplementary information

Supplementary Method and Figure 1

Supplementary Table 1

Supplementary Table 2

Supplementary Table 3
