# Supplementary material for: Peripheral artery disease: an underdiagnosed condition in familial hypercholesterolemia? A systematic review
Source: Endocrine. 2024 Mar 8;85(1):122–33. doi: 10.1007/s12020-024-03763-x (PMC11246299; doi:10.1007/s12020-024-03763-x)

**Peripheral artery disease: an underdiagnosed condition in familial hypercholesterolemia? A systematic review.**

Elisa Acitelli, MD^1^; Alexis F. Guedon, MD^2^; Sara De Liguori, MD^1^; Antonio Gallo, MD, PhD^3^*; Marianna Maranghi, MD,PhD^1^*

^1^Department of Translational and Precision Medicine, Sapienza University of Rome, Rome Italy

**^2^** Sorbonne Université, APHP, Service de Médecine Interne, Département Hospitalo-Universitaire Inflammation Immunopathologie Biothérapie (DMUi3), Paris, France

^3^Sorbonne Université, INSERM UMR1166, Lipidology and cardiovascular prevention Unit, Department of Nutrition, APHP, Hôpital Pitié-Salpètriêre, 47/83 boulevard de l’Hôpital, F-75013, Paris, France

* Equal contribution

Corresponding author: Marianna Maranghi (marianna.maranghi@uniroma1.it)

**Supplementary Methods**

Research Algorithm

Potential trials were identified from MEDLINE and Embase. The following algorithms were used in MEDLINE:("Hyperlipoproteinemia Type II"[Mesh] OR "familial hypercholesterolemia" OR "familial hypercholesterolaemia") AND ("Peripheral Arterial Disease"[Mesh] OR "peripheral artery disease" OR "lower extremity arterial disease") AND 2013/01/01:2023/11/14[Date - Publication]) and in Embase:('peripheral arterial disease'/exp OR 'peripheral arterial disease') AND ('familial hypercholesterolemia'/exp OR 'familial hypercholesterolemia') AND [article]/lim AND [embase]/lim AND [2013-2023]/py AND ('cohort analysis'/de OR 'controlled study'/de OR 'prospective study'/de OR 'randomized controlled trial'/de OR 'retrospective study'/de) AND [01-01-2013]/sd NOT [15-11-2023]/sd.

**Supplementary Figure 1**. PRISMA Flow Diagram for Study Selection


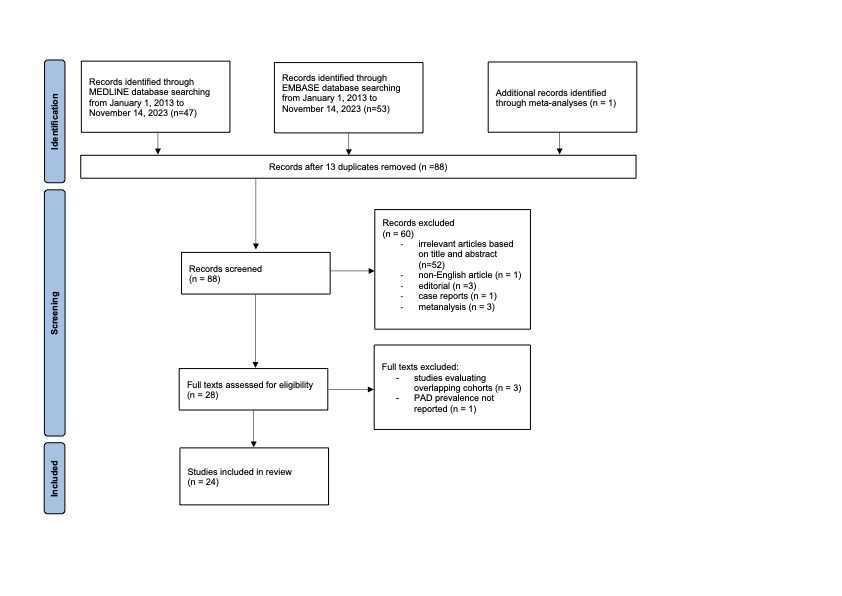

Supplement: Supplementary file 1 — Supplementary Method and Figure 1 [file 12020_2024_3763_MOESM1_ESM.docx]
