# Supplementary material for: Peripheral artery disease: an underdiagnosed condition in familial hypercholesterolemia? A systematic review
Source: Endocrine. 2024 Mar 8;85(1):122–33. doi: 10.1007/s12020-024-03763-x (PMC11246299; doi:10.1007/s12020-024-03763-x)
Supplement: Supplementary file 2 — Supplementary Table 1 [file 12020_2024_3763_MOESM2_ESM.docx]

**Supplementary Table 1**. Lipid parameters and cardiovascular disease of FH subjects in studies evaluating PAD prevalence.

| **Year,**  **Journal,**  **First Author**  **Country** | **Population (n)** | **Genotype** | **Lipid profile (mg/dl)** | | | | | **Previous ASCVD prevalence: n (%)** |
| --- | --- | --- | --- | --- | --- | --- | --- | --- |
|  |  |  | **Overall population** | ***Subgroup 1** | ***Subgroup 2** | ***Subgroup 3** | ***Subgroup 4** |  |
| 2016,  Arterioscler Thromb Vasc Biol,  L Pérez de Isla,  Spain [11] | 2 752 | LDL-C receptor null mutation 993 (36.1%), LDL-C receptor defective mutation 1140 (41.4%), LDL-C receptor UKF mutation 529 (19.2%), ApoB mutation 90 (3.3%) | TC 237  (205–280.8)  LDL-C 165 (138.6–207.8)  HDL-C 49  (41–57.2)  Non-HDL-C 185 (155–229.5)  TG 84 (63–116.3)  Lp(a) 22.6 (8.8–55.6)  Apo-B 109 (91–133)  Apo-AI 135 (118–153) |  |  |  |  | Overall 260 (9.4)  [angina 205 (7.5), AMI 178 (6.5), Coronary angioplasty/stent 139 (5.0), CABG 110 (4.0), any atherosclerotic coronary artery disease 325 (11.8), Stroke 21 (0.8), TIA 23 (0.8), Carotid stent 8 (0.3),Carotid surgery 7 (0.3) Peripheral artery revascularization 14 (0.5), any atherosclerotic cerebrovascular disease 49 (1.8),  any atherosclerotic peripheral artery disease 39 (1.4)] |
| 2015, Atherosclerosis,  C Pereira,  Brazil [12] | 202 | NA | TC: 336 ± 87 | TC 332 ± 84.4  LDL-C 254 ± 82  HDL-C 46 ± 12  TG 135 ± 61 | TC 357 ± 99  LDL-C 275 ± 100  HDL-C 46 ± 13  TG 150 ± 53 |  |  | Overall 57 (28.2) |
| 2022,  J Am Heart Assoc,  S Funabashi,  Japan [13] | 370 | LDL-C receptor: 218 (59%), PCSK9 25 (6.7%), LDL-C receptor and PCSK9: 17 (4.6%) | NA | °LDL-C 124 ± 50.2  HDL-C 60 ± 14.4  TG 78 (58–113)  Lp(a) 14.9 (6.9–30.9) | °LDL-C 90 ± 37.8  HDL-C 51 ± 14.4  TG 93 (63–134)  Lp(a) 18.4 (9–42.7) | °LDL-C 91 ± 32.8  HDL-C 44 ± 10.7  TG 106 (87–156)  Lp(a) 49 (17.6–70.7) |  | NA |
| 2021, Atherosclerosis, AJ Vallejo-Vaz, Multicenter Scandinavian countries [14] | 152 | NA | NA | TC 284.6 ± 19.7  LDL-C 212.3 ± 18.1  HDL-C 44.4 ± 9.6  TG 137.3 (110.7-  168.3) | TC 287.3 ± 17  LDL-C 213.4 ± 15  HDL-C 47.1 ± 10.8  TG 132.8 (97.4-  168.3) |  |  | Overall 152 (100)  [Myocardial infarction 106 (69)  CABG 7 (4.6)  PTCA 3 (1.9)] |
| 2018,  J Clin Endocrinol Metab,  F Emanuelsson,  Denmark [15] | 7 109 | LDL-C receptor W23X, W66G, W556S, or ApoB R3500Q mutation: 169 (2.3%) | NA | TC 293 (271–313)  LDL-C 201 (193–215)  HDL-C 54 (46–66)  TG 158 (114–229)  Lp(a) 13.8 (8.6–49.3) | TC 336 (290–372)  LDL-C 256 (205–277)  HDL-C 54 (46–69)  TG 150 (97–220)  Lp(a) 17.6 (7.8–43) |  |  | NA |
| 2019,  J Clin Med, L Masana,  Spain [16] | 12 823 | NA | NA | TC 257.9 ± 59.5  LDL-C 177.6± 55.5  HDL-C 56.3 ± 13.8  Non-HDL-C 201.7 ± 59.3  TG 149.1 ± 88 | TC 223.9 ± 5  LDL-C 147.1 ± 46  HDL-C 51.6 ± 13  Non-HDL-C 172.3 ± 51.3  TG 150.6 ± 83.1 |  |  | Overall 2 202 (17.1)  [CAD 1 032 (46.9)  stroke 502 (22.8)] |
| 2018, Atherosclerosis,  YX Cao,  China [17] | 151 | LDL-C receptor 104 (68.9%), Apo-B 28 (18.5%) | TC 288.1 ± 102.8  LDL-C 217.3 ± 92  HDL-C 42.1 ± 13.1  TG 170 ± 197.5  Lp(a) 27.9 (12.7-61) |  |  |  |  | NA |
| 2019,  NMCD,  A Mattina,  France [18] | 154 | NA | TC 252.1 ± 67.6  LDL-C 175.9 ± 64.5  HDL-C 56.4± 20.1  TG 104.5 ± 62.8  Lp(a) 27 (10-54) |  |  |  |  | Primary prevention population |
| 2017,  Arterioscler Thromb Vasc Biol, AJ Amor,  Spain [19] | 240 | NA  . | TC: 359 (324–424)  LDL-C 286 (246–346)  HDL-C 49 (43–62)  TG 112 (78–161)  Lp(a) 22 (10–52)  Apo-B 188 (162–219)  Apo-AI 134 (119–154) |  |  |  |  | Overall 33 (14) |
| 2018,  Am J Cardiol,  PA McCullough,  Multicountry [20] | 1 257 | NA | NA | LDL-C 148.1 ± 53  HDL-C 49.3 ± 13.9  Non-HDL-C 175.1 ± 57.1  TG 119.5 (86-158)  Lp(a) 37.3 (12-89)  Apo-B 117.9 ± 31.8 | LDL-C 151 ± 57.1  HDL-C 48.9 ± 13.5  Non-HDL-C 178.4 ± 60.7  TG 119 (93-167)  Lp(a) 31.4 (11.5-96)  Apo-B 119.2 ± 34.6 | LDL-C 155.9 ± 55.5  HDL-C 52.1 ± 14.9  Non-HDL-C 181 ± 59.5  TG 109.9 (82-149)  Lp(a) 22.2 (9-58)  Apo-B 118.4 ± 33.2 | LDL-C 148.3 ± 44.9  HDL-C 51 ± 15  Non-HDL-C 172.4 ± 47.9  TG 101 (76-142)  Lp(a) 18 (5-57.2)  Apo-B 114.2 ± 26.4 | Overall 575 (45.7)  [Acute coronary syndrome 356 (61.9), coronary revascularization procedure 389 (67.6), other clinically significant coronary heart disease 285 (49.5), ischemic stroke 44 (7.6)] |
| 2022, Atheroscler Plus, WT Fonzar, Brazil [21] | 92 | pathogenic or likely pathogenic 28 (30%) , VUS 17 (19%), no mutation, benign or not performed 47 (51%) | TC 233 (186-296)  LDL-C 141 (104-202)  HDL-C 54 (43 - 65)  Non-HDL-C 173 (131- 237)  TG 135 (96-176)  Lp(a) 23 (8-53)  Apo-B 114 (88-142)  Apo-AI 149 (129-151) |  |  |  |  | NA |
| 2020, Oman Med J, K Al-Waili, Oman [22] | 439 | Confirmed FH-related mutation 33.9 % | NA | TC 293.9 ± 30.9  LDL-C 220.4 ± 27 | TC 355.7 ± 73.4  LDL-C 286.1 ± 69.6 |  |  | NA  [Angina 53 (12)  CAD 98 (22.3)  Myocardial infarction: 53 (12)  premature CBVD: 13 (2.9)] |
| 2022,  Endocrine,  P Anagnostis,  Greece [23] | 541 | NA | NA | TC 324 ± 70  LDL-C 240 ± 70  HDL-C 56 ± 18  Non-HDL-C 268 ± 72  TG 118 (77–167)  Lp(a) 6.4 (3–9.7)  Apo-B 142 ± 50  Apo-AI 147 ± 34 | TC 331 ± 74  LDL-C 250 ± 73  HDL-C 54 ± 15  Non-HDL-C 278 ± 74  TG 123 (87–165)  Lp(a) 22.4 (16–29.1)  Apo-B 159 ± 52  Apo-AI 146 ± 31 | TC 335 ± 79  LDL-C 253 ± 80  HDL-C 55 ± 21  Non-HDL-C 279 ± 80  TG 117 (85–165)  Lp(a) 77  (55–102)  Apo-B 160 ± 55  Apo-AI 148 ± 30 |  | NA  **Subgroup 1** 17 (9.4)  [Premature ASCVD 15 (8.5)  CAD 15 (8.3)  Premature CAD 15 (8.3)  Stroke 4 (2.2)]  **Subgroup** **2** 29 (16.1)  [Premature ASCVD 24 (13.4)  CAD 22 (12.2)  Premature CAD 20 (11.1)  Stroke 6 (3.3)]  **Subgroup 3** 37 (20.6)  [Premature ASCVD 35 (19.8)  CAD 29 (16.1)  Premature CAD 28 (15.6)  Stroke 9 (5)] |
| 2018,  Circ J,  B Zafrir  Israel [24] | 1 690 | NA | LDL-C 181 ± 69  HDL-C 49.8 ± 14 |  |  |  |  | Overall 248 (14.6) |
| 2019, Atherosclerosis, PB Duell  US [25] | 1 900 | NA | TC 225.2 ± 74.5  LDL-C 145.3 ± 67.5  HDL-C 54.2 ± 17.4  TG 145 ± 93.7  Lp(a) 28 (10-83.5) |  |  |  |  | Overall 704 (37.1) |
| 2021, Med Sci Monit, Matta A.  France [26] | 123 | NA | TC 361 ± 83  LDL-C 277 ± 78  HDL-C 52 ± 13  TG 168 ± 69 |  |  |  |  | Overall 94 (76.4)  [CAD 82 (66.7), ischemic stroke 3 (2.4)] |
| 2022, Front Genet, V Todorovova, Chzechia [27] | 1 236 | patients  genetically tested 1008, patients with mutations of ApoB-100 154, patients supposed to have mutations in LDL-C receptor gene 854 | NA | ✵TC 346.1 ± 75.4  LDL-C 250.9 ± 74.2  HDL-C 64.5 ± 17.7  TG 160.3 ± 100  Lp(a) 56 ± 74  Apo-B 176 ± 56 |  |  |  | NA  [CAD (MI included) (9.6)  Stroke (2.5)] |
| 2019, Turk J Endocrinol Metab, MA Eren, Turkey [28] | 267 | NA | TC 306.8 ± 57.6  LDL-C 219.5 ± 49.4  HDL-C 48.3 ± 13.1  TG 136 ± 55.9 |  |  |  |  | NA  [CBVD 11 (4.1),  CAD 32 (12)] |
| 2018, J Atheroscler Thromb, T Teramoto,  Japan [29] | 3 495 | NA | LDL-C 145.8 ± 46.6 |  |  |  |  | NA  [CAD 584 (16.7)  stroke 42 (1.2)] |

LDL-C: low-density lipoprotein cholesterol; UKF mutation: mutation without functionality assays; ApoB: Apolipoprotein B; TC: total cholesterol; HDL-C: high-density lipoprotein cholesterol; Non-HDL-C: Non High-density lipoprotein cholesterol; TG: triglycerides; Lp(a): lipoprotein(a); ApoAI: apolipoprotein AI; ASCVD: atherosclerotic cardiovascular disease; AMI: acute myocardial infarction; CABG: coronary artery bypass graft; TIA, transient ischemic accident; NA: not available; PCSK9:proprotein convertase subtilisin/kexin type 9; PTCA: percutaneous transluminal coronary angioplasty; CAD: Coronary Artery Disease; VUS: variant of uncertain significance; MI: myocardial infarction; CBVD: cerebrovascular disease.

Distribution of ASCVD based on type of disease and vascular district involved are reported between square brackets.

Some data were extracted from those reported in the original articles.

**￪**Data are presented as mean ± standard deviation or median (IQR)

* Lipid profile is reported as stratified by subgroups if data about the overall population was not available

°Data were available only for subjects on lipid lowering treatment

✵patients were tested randomly for lipids
