# Supplementary material for: Peripheral artery disease: an underdiagnosed condition in familial hypercholesterolemia? A systematic review
Source: Endocrine. 2024 Mar 8;85(1):122–33. doi: 10.1007/s12020-024-03763-x (PMC11246299; doi:10.1007/s12020-024-03763-x)
Supplement: Supplementary file 3 — Supplementary Table 2 [file 12020_2024_3763_MOESM3_ESM.docx]

**Supplementary Table 2**. Lipid parameters and cardiovascular disease of FH subjects in studies evaluating PAD incidence.

| **Year,**  **Journal,**  **First Author**  **Country** | **Population (n)** | **Genotype** | **Lipid profile (mg/dl)** | | | | | **Previous ASCVD prevalence: n (%)** | **ASCVD incidence: n (%)** |
| --- | --- | --- | --- | --- | --- | --- | --- | --- | --- |
|  |  |  | **Overall population** | ***Subgroup 1** | ***Subgroup 2** | ***Subgroup 3** | ***Subgroup 4** |  |  |
| 2021,  JACC Asia,  S Funabashi  Japan [30] | 380 | LDL-C receptor variants 57%; PCSK9 variants 9%; LDL-C receptor and PCSK9 variants 4% | NA | LDL-C 212 ± 57  HDL-C 60 ± 14  TG 91 (61-140)  Lp(a) 16.7 (10.4-26.8) | LDL-C 283 ± 64  HDL-C 53 ± 13  TG 118 (82-162)  Lp(a) 22.7 (11.7-41.2) |  |  | Primary prevention population | Cardiac-death 1 (0.3)  CAD 31 (8.2)  Coronary revascularization 29 (7.6)  Ischemic stroke 11 (2.9) |
| 2022,  Eur J Prev Cardiol,  LJ Mundal  Norway [31] | 3 162 | NA | NA |  |  |  |  | NA | NA |
| 2022, Atherosclerosis, J Ferrieres  France [32] | 3 202 | NA | NA | TC 264 ± 82  LDL-C 187 ± 76  HDL-C 55 ± 17  TG 122 ± 230 | TC 232 ± 85  LDL-C 153 ± 76  HDL-C 50 ± 14  TG 140 ± 91 |  |  | Overall 717 (22.4)  [Premature cardiovascular event 570 (79)] | CVD events 387 (9)  AMI 155 (40),  Aortic valve replacement 28 (7)  Unstable angina 102 (26), other acute heart disease 11 (3), stroke 24 (6), TIA 20 (5) |
| 2019, Atherosclerosis,  B Iyen  UK [33] | 14 097 | NA | TC 359.6 ± 100.5 LDL-C 221.2 ± 81.2  TG 186 (115.1 - 310) |  |  |  |  | NA | Coronary heart disease 3 545, Stroke/TIA 764 |
| 2018, AM Galema-Boers, J Clin Lipidol  Netherlands  [34] | 821 | LDL-C receptor mutations 552 patients (67%)  ApoB mutation 65 patients (8%) | TC 209 ± 62  LDL-C 139 ± 58  HDL-C 54 ± 15  TG 115 ± 62 |  |  |  |  | Overal: 95 (12) | CVD events 102 (12)  Myocardial infarction 36 (35), angina pectoris 12 (12),  PCI/CABG 27 (27)  TIA 13 (13), stroke 10 (10) |

LDL-C: low-density lipoprotein cholesterol; TC: total cholesterol; HDL-C: high-density lipoprotein cholesterol; TG: triglycerides; Lp(a): lipoprotein(a); ApoB: Apolipoprotein B; ASCVD: atherosclerotic cardiovascular disease; AMI: acute myocardial infarction; CABG: coronary artery bypass graft; PCI: percutaneous coronary intervention; TIA, transient ischemic accident; NA: not available; PCSK9:proprotein convertase subtilisin/kexin type 9; PTCA:percutaneous transluminal coronary angioplasty; CAD: Coronary Artery Disease; CVD: cardiovascular disease

Distribution of ASCVD based on type of disease and vascular district involved are reported between square brackets.

Some data were extracted from those reported in the original articles.

**￪**Data are presented as mean ± standard deviation or median (IQR)

*Lipid profile is reported as stratified by subgroups if data about the overall population was not available.
