# Supplementary material for: Peripheral artery disease: an underdiagnosed condition in familial hypercholesterolemia? A systematic review
Source: Endocrine. 2024 Mar 8;85(1):122–33. doi: 10.1007/s12020-024-03763-x (PMC11246299; doi:10.1007/s12020-024-03763-x)
Supplement: Supplementary file 4 — Supplementary Table 3 [file 12020_2024_3763_MOESM4_ESM.docx]

**Supplementary Table 3.** ICD-8, ICD-9 and ICD-10 codes used for PAD definition in the analyzed literature

| **ICD-8** | 440 | Arteriosclerosis |
| --- | --- | --- |
|  | 441 | Aortic aneurysm (non-syphilitic) |
|  | 443.99 | Intermittent arterial claudication |
|  | 445 | Gangrene |
| **ICD-9** | 440 | Atherosclerosis |
|  | 443 | Other peripheral vascular diseases |
|  | 4439 | Peripheral vascular disease, unspecified |
|  | 44020 | Atherosclerosis of native arteries of the extremities, unspecified |
|  | 44021 | Atherosclerosis of native arteries of the extremities, with intermittent claudication |
|  | 44022 | Atherosclerosis of native arteries of the extremities, with pain at rest |
|  | 44023 | Atherosclerosis of native arteries of the extremities, with ulcers |
|  | 44024 | Atherosclerosis of native arteries of the extremities, with gangrene |
|  | 44029 | Other atherosclerosis of native arteries of the extremities |
|  | 44030 | Atherosclerosis of unspecified bypass graft of the extremities |
|  | 44031 | Atherosclerosis of autologous venous bypass graft of the extremities |
|  | 44032 | Atherosclerosis of non-autologous venous bypass graft of the extremities |
|  | 44422 | Embolism and thrombosis of arteries of the lower extremities |
|  | 44489 | Other arterial embolism and thrombosis |
| **ICD-10** | I70 | Atherosclerosis |
|  | I70.2 | Atherosclerosis of native arteries of the extremities |
|  | I70.8 | Atherosclerosis of other arteries |
|  | I70.9 | Other and unspecified atherosclerosis |
|  | I71 | Aortic aneurysm and dissection |
|  | I72 | Other aneurysm |
|  | I73 | Other peripheral vascular diseases |
|  | 173.8 | Other specified peripheral vascular diseases |
|  | I73.9 | Intermittent arterial claudication |
|  | I74.3 | Embolism and thrombosis of arteries of the lower extremities |
